# The Halophilic Bacterium *Paracoccus haeundaensis* for the Production of Poly(3-Hydroxybutyrate-*co*-3-Hydroxyvalerate) from Single Carbon Sources

**DOI:** 10.4014/jmb.2305.05025

**Published:** 2023-11-13

**Authors:** Seon Min Kim, Hye In Lee, Seung Won Nam, Deok Hyeon Jin, Gwi-Taek Jeong, Soo-Wan Nam, Brendan Burns, Young Jae Jeon

**Affiliations:** 1 Department of Microbiology, College of Natural Sciences, Pukyong National University, Busan 48513, Republic of Korea; 2 School of Marine and Fisheries Life Science, Pukyong National University, Busan 48513, Republic of Korea; 3 Bioresources Collection and Research Team, Nakdonggang National Institute of Biological Resources, Sangju 37242, Republic of Korea; 4 Department of Biotechnology, Pukyong National University, Busan 48513, Republic of Korea; 5 Department of Smart Bio-Health, Dong-eui University, Busan 47340, Republic of Korea; 6 Biomedical Engineering and Biotechnology Major, Division of Applied Bioengineering, College of Engineering, Dong-eui University, Busan 47340, Republic of Korea; 7 School of Biotechnology & Biomolecular Science, The University of New South Wales, Sydney, NSW 2052, Australia

**Keywords:** *Paracoccus haeundaensis*, nitrogen-limited conditions, polyhydroxyalkanoates, poly(3-hydroxybutyrate), poly(3-hydroxybutyrate-*co*-3-hydroxyvalerate), batch fermentation

## Abstract

The study objective was to evaluate the potential production of polyhydroxyalkanoates (PHAs), a biodegradable plastic material, by *Paracoccus haeundaensis* for which PHA production has never been reported. To identify the most effective nitrogen-limited culture conditions for PHAs production from this bacterium, batch fermentation using glucose concentrations ranging from 4 g l^-1^ to 20 g l^-1^ with a fixed ammonium concentration of 0.5 g l^-1^ was carried out at 30°C and pH 8.0. A glucose supplement of 12 g l^-1^ produced the highest PHA concentration (1.6 g l^-1^) and PHA content (0.63 g g^-1^) thereby identifying the optimal condition for PHA production from this bacterium. Gas chromatography-mass spectrometry analysis suggests that *P. haeundaensis* mostly produced copolymer types of poly(3-hydroxybutyrate-*co*-3-hydroxyvalerate) [P(3HB-*co*-3HV)] from glucose concentrations at 12 g l^-1^ or higher under the nitrogen-limited conditions. When several other single carbon sources were evaluated for the most efficient PHA production, fructose provided the highest biomass (2.8 g l^-1^), and PHAs (1.29 g l^-1^) concentrations. Results indicated that this bacterium mostly produced the copolymers P(3HB-*co*-3HV) from single carbon sources composing a range of 93–98% of 3-hydroxybutyrate and 2–7% of 3-hydroxyvalerate, whereas mannose-supplemented conditions produced the only homopolymer type of P(3HB). However, when propionic acid as a secondary carbon source were supplemented into the media, *P. haeundaensis* produced the copolymer P(3HB-*co*-3HV), composed of a 50% maximum monomeric unit of 3-hydroxyvaleric acid (3HV). However, as the concentration of propionic acid increased, cell biomass and PHAs concentrations substantially decreased due to cell toxicity.

## Introduction

Plastics obtained using fossil resources have a valuable role in our daily life and are involved in various purposes including the materials used in packaging, medicine, agriculture, automobiles, and food industries [1]. Despite their wide range of applications, their very low biodegradability has caused serious environmental concerns [1]. Furthermore, the current disposal methods for petro-plastic waste that rely on recycling, landfill, and incineration, do not provide fundamental solutions due to low recycling rates and the generation of additional pollutants such as dioxin during waste treatments [1, 2]. Therefore, biodegradable plastics have been strategically adopted in the production of commercial goods as an effective means to minimize the hazardous potential triggered by microplastics (MPs). The diverse types of biodegradable polymers are currently produced from renewable resources [3]. However, their high production costs are one of the major barriers satisfying current plastic market demand [4].

Polyhydroxyalkanoates (PHAs) are biodegradable bioplastics produced by a variety of microorganisms, under limited nutrient conditions, as intracellular energy storage compounds [5, 6]. Because PHAs are easily degraded by many microorganisms in natural environments, the bioplastics are considered as ecofriendly-biodegradable polymers [7]. In addition, since their mechanical properties and melting points can be controlled by regulating the type and ratio of PHA monomers, PHAs are used as promising alternatives in various industries such as medicine, food, and energy. Currently, about 150 different types of PHA monomers have been identified. According to the length of the monomer, PHAs can be classified as short-chain length PHAs (SCL-PHAs) which contain C_3_-C_5_ monomers, medium-chain length PHAs (MCL-PHAs) which contain C_6_-C_14_ monomers, and long-chain length PHAs (LCL-PHA) which contains C_15_ or higher carbon monomers [8]. SCL-PHAs are similar to conventional plastics such as polypropylene (PP) and polyethylene (PE), but their stiff and brittle mechanistic properties due to their high crystallinity and poor mechanical properties in terms of Young's modulus and tensile strength are one of the major disadvantages preventing wide application [9]. In particular, poly(3-hydroxybutyrate) [P(3HB)] is the most common homopolymer of SCL-PHA and is produced by various types of bacteria. However, different hydroxyalkanoate monomeric units incorporated into 3HB by the formation of copolymer show an improvement in the mechanical properties. Among them, poly(3-hydroxybutyrate-*co*-3-hydroxyvalerate) [P(3HB-*co*-3HV)], a copolymer composed of various molar ratios of both 3HB and 3HV monomeric units, has superior advantages for industrial application because it is not brittle like PHB and can serve as a preferred alternative [10].

Recently, halophiles are attracting considerable interest as a promising and cost-effective producer of the copolymer of PHA [11]. The advantages of halophilic PHA producers for use in industrial sectors have been highlighted in terms of production of type of copolymer PHA such as P(3HB-*co*-3HV) produced from a single carbon source, but also in terms of reducing the production costs associated with cheaper raw materials, water use and purification steps [12, 13]. For P(3HB-*co*-3HV) synthesis and production in the lab and industry, in general the precursor of 3HV such as propionic acid is required to the culture medium. However, such precursor is a relatively expensive chemical. Therefore, various research strategies have been adapted to increase such copolymers with high molar ratio of 3HV [14, 15].

*Paracoccus haeundaensis* is a halophilic marine bacterium that is relatively well-documented for astaxanthin production [16-18]. To our best knowledge, the potential PHA production of this bacterium has not yet been documented. Therefore, the use of a marine halophilic bacterium, such as *P. haeundaensis*, for the potential production of PHA was investigated through the prediction of the PHA synthesis pathway *in silico* in this bacterium and batch fermentation experiments using various single carbon sources were performed under nitrogen-limited conditions.

## Materials and Methods

### Bacterial Strain and Culture Media

The strain used in this study, *Paracoccus haeundaensis* BC74171^T^ (=KCCM 10460^T^ =LMG P-21903^T^), was obtained from the Korean Culture Center of Microorganisms (KCCM), Republic of Korea. This bacterium was cultivated at 30°C in M9 medium with a final concentration per liter of 7.52 g Na_2_HPO_4_·2 H_2_O (Duksan, Republic of Korea), 3.0 g KH_2_PO_4_ (Sigma, USA), 30 g NaCl (Samchun, Republic of Korea), 0.5 g NH_4_Cl (Daejung, Republic of Korea), 0.25 g MgSO_4_·7 H_2_O (Shinyo Chemicals, Japan), 40 mg CaCl_2_·2H_2_O (Duchefa Biochemie, Netherlands), 1 μg biotin (Duchefa Biochemie), 1 μg thiamin (Hayashi, Japan), and 10 ml trace element solution (pH 7.5) with a final concentration per liter of 5 g EDTA (Duchefa Biochemie), 0.83 g FeCl_3_·6H_2_O (Daejung), 84 mg ZnCl_2_ (Daejung), 13 mg CuCl_2_·2H_2_O (Duksan), 10 mg CoCl_2_·2H_2_O (Junsei, Japan), 10 mg H_3_BO_3_ (Shinyo Chemicals), and 1.6 mg MnCl_2_·4H_2_O (Junsei, Japan). To promote the growth of *P. haeundaensis*, 1 ml of multivitamin containing pyridoxine hydrochloride (Junsei), riboflavin (Duchefa Biochemie), pantothenic acid (Junsei), and nicotinic acid at 1 mg l^-1^, respectively, were supplemented into M9 media (hereafter mentioned as M9MV). The pH of the medium was adjusted to 8.0 using 5 M sodium hydroxide. For sub-culturing purposes, M9MV agar plates containing 8 g l^-1^ glucose and 15% agar were used.

### *In silico* Prediction of PHA Metabolic Pathways in *P. haeundaensis*

The pathways and genes involved in the production of PHA in *P. haeundaensis* were identified by analyzing genome sequences available at GenBank (assembly accession: GCA_006239215.1) and the Kyoto Encyclopedia of Genes and Genomes (KEGG) database. Pathway prediction for the strain genome was also achieved using BlastKOALA [19, 20].

### PHA Production Using Various Carbon Sources

A single colony of *P. haeundaensis*, taken from a culture inoculated onto an M9MV plate, was inoculated into 100 ml Erlenmeyer flasks containing 30 ml M9MV medium supplemented with 8 g l^-1^ glucose and subsequently incubated at 30°C in a shaking incubator (Vision Scientific, Westland) at 160 rpm until it reached an optical density (OD_600_) of 2.0, and this was then used as the inoculum in the batch fermentation. All batch fermentation was carried out using 10 % (v/v) inoculum in 500 ml Erlenmeyer flasks containing 250 ml of M9MV media at a temperature of 30°C and shaken at 160 rpm to evaluate PHA production. To produce nitrogen-limited conditions, NH_4_Cl was supplemented at 0.5 g l^-1^. To determine carbon excess conditions for PHA production, predetermined glucose concentrations ranging from 4 g l^-1^ to 20 g l^-1^ were supplemented into M9MV. The carbon and nitrogen mass ratio (C/N ratio, g/g) was calculated using a previously reported method [21].

To determine which carbon sources could influence PHA production in this bacterium, various pre-determined carbon sources including glucose (Junsei), fructose (CHAMCHUN, Republic of Korea), lactose (Difco, France), galactose (CHAMCHUN), sucrose (Sigma, USA), glycerol (Sigma), and xylose (Junsei) at 12 g l^-1^, respectively, were used to supplement the M9MV media.

### PHA Production Using Propionic Acid as a Secondary Carbon Source

To improve the monomeric unit ratio of 3-hydroxyvalerate in the PHAs produced by *P. haeundaensis*, propionic acid, which was identified as a precursor of 3-hydroxyvalerate in the predicted *in silico* PHA biosynthetic pathway of *P. haeundaensis*, was additionally supplemented to the M9MV culture medium as a secondary carbon source. Together with pre-determined glucose concentrations, propionic acid was supplemented using concentrations ranging from 0 g l^-1^ to 4 g l^-1^.

### PHAs Quantification

The concentration of PHAs was determined by gas chromatography using a previously reported methanolysis method [22]. Harvested cells from batch fermentation experiments were washed with deionized water three times. The cell pellet was lyophilized using a freeze-dry machine and stored in 15 ml Pyrex glass tubes until required. The dried cells were then treated with 1 ml chloroform (Junsei) and 1 ml methanol (Sigma) containing 15 % (v/v) sulfuric acid and the tubes were capped with PTFE seal caps and incubated at 100°C in a water bath for 2.5 h. After cooling on ice for 10 min, 1 ml of chloroform containing 0.2 % (v/v) methyl benzoate (Sigma) as an internal standard and 1 ml of deionized water were added to the tubes and vortexed for 30 sec. The lower solvent phase was collected and used for PHA quantification. Poly[(R)-3-hydroxybutyric acid] (Sigma) and P(3HB-*co*-3HV) (Sigma) were used as standard chemicals for the calibration of 3HB and 3HV monomeric unit quantification, respectively. Identical experimental procedures to those used above for the sample preparation for PHA quantification were used for the standard chemicals. Finally, all samples and standards were filtered through PTFE/H hydrophobic syringe filters (CHMLAB, 0.2 μm) and subjected to PHA quantification via GC or GC/MS.

### Transmission Electron Microscopy (TEM)

Cells were prefixed in a 2% (v/v) glutaraldehyde in 0.1 M phosphate buffer (pH 7.0) for one day at 4°C. The glutaraldehyde-fixed cells were washed three times in 0.1 M phosphate buffer (pH 7.0) and post-fixed in 1% (W/V) OsO4 for 1 h at 4°C. The fixed cells were then rinsed three times with distilled water. Dehydration was carried out at 4°C using a graded ethanol series of 50%, 60%, 70%, 80%, and 90% for 10 min each and three times with 10 min changes of pure ethanol. Pellets were then brought to room temperature and transferred through propylene oxide two times for 20 min each with 50% and 75% Spurr’s embedding resin [12, 23] in propylene oxide for 1 h each and 100% overnight. The following day, pellets were transferred to new pure resin and polymerized at 70°C. Blocks were thin-sectioned on a PT-X ultramicrotome (RMC Products, Boeckeler Instruments, USA). Sections of 70 nm thickness were collected on slot copper grids, stained with UranyLess solution (Electron Microscopy Sciences, USA) and Reynold’s lead citrate [12, 24] and observed and photographed using a JEM-2100F transmission electron microscope operated at 120 kV (Jeol, Japan).

### Analytical Techniques

Cell growth was determined by optical density at 600 nm using a spectrophotometer (Unicam UV-VIS GB/UV 540, USA). For the kinetic evaluation of cell biomass concentrations, cell dry weight (CDW) was determined using a gravimetric method. A ten-milliliter culture broth was collected and centrifuged at 9,000 rpm for 10 min. The harvested cells were washed with deionized water three times and dried at 105°C in a dry oven (Daihan Scientific, Republic of Korea) overnight.

To determine carbon source consumption by *P. haeundaensis*, glucose, fructose, lactose, galactose, glycerol, xylose, mannose concentrations were quantified using a previously reported HPLC method [25]. For the analysis, a HPLC (UltiMate 3000, Thermo Fisher Scientific, USA) equipped with an Aminex HPX-87H (BioRad, USA) column and refractive index detector was used. As a mobile phase, 5 mM H_2_SO_4_ was prepared and used. To analyze carbon concentrations, 20 ml of each sample was injected into the HPLC system using 5 mM H_2_SO_4_ at a flow rate of 0.6 ml min^-1^ for 30 min, with an oven temperature of 50°C.

Quantification of PHA after methanolysis was carried out using a gas chromatograph (Agilent, Agilent 6890N) and a flame ionization detector [22, 26]. An Aligent DB-WAX Ultra Inert capillary column (30 m × 0.25 mm × 0.25 μm) was used to analyze the PHA monomers generated from methanolysis. The analysis was performed using an injection temperature of 250°C with a split injection mode. The column temperature was programmed as follows: 2 min hold at 80°C, 10°C min^-1^ to 245°C and hold for 1 min, pressure of 14.9 psi, total flow of 26.5 ml min^-1^, injection split 20:1, and split flow 22.7 ml min^-1^.

Gas chromatography-mass spectrometry (Shimadzu, GCMS QP-2010Ultra) was further used to confirm the monomeric compositions of the PHAs. To analyze the PHA monomers generated from methanolysis, a capillary column (DB-5MS Ultra, 30 m × 0.25 mm × 0.25 μm) was used. The analysis was conducted using an injection temperature set at 260°C with a split injection mode. The column temperature was programmed as follows: 2 min hold at 80°C, 10°C min^-1^ to 120°C, 45°C min^-1^ to 270°C and hold for 3.67 min. Injection temperatures of 260°C, pressure of 65.2 kPa, total flow of 104.0 ml min^-1^, injection split 100:1, and split flow 3.0 ml min^-1^. The PHAs content (g g^-1^) was calculated as a cell dry weight (CDW) on a mass basis. The PHA monomer composition (mol%) was expressed as a molar percentage of the total number of monomers.

## Results and Discussion

### Nutritional Requirements of *P. haeundaensis*

**Fig. 1:**
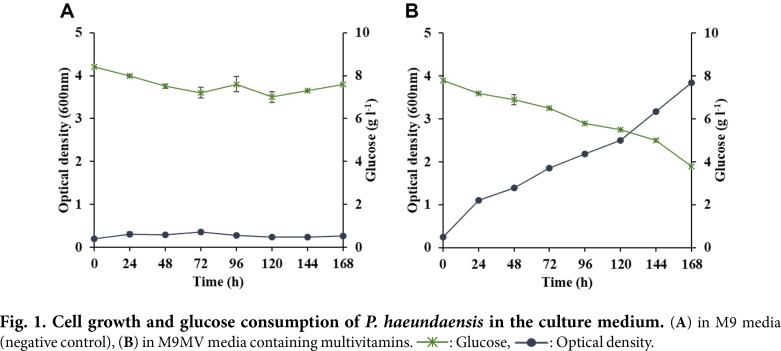


Before investigating the PHAs production potential of *Paracoccus haeundaensis*, M9 broth supplemented with 8g l^-1^ of glucose, which provided chemically defined nutritional conditions, was chosen to investigate cell growth performance. The results, as shown in Fig. 1A, confirm that throughout the 168 h of culturing time, the 8 g l^-1^ of glucose remained unconsumed and the cell biomass optical density (OD_600nm_) only reached 0.1, indicating that M9 broth required additional growth factors for bacterial cell growth under this condition. To identify the additional growth requirements, several studies were carried out with various combinations of vitamins and other growth factors (data not shown). One particular experiment (Fig. 1B), in which M9 broth was supplemented with several vitamins (referred to from here as M9MV broth) including pyridoxine, riboflavin, pantothenic acid, and nicotinic acid, showed a significant improvement in cell growth. Although 4 g l^-1^ of residual glucose remained unconsumed at the end of cultivation, optical densities reached 4.0, indicating that *P. haeundaensis* is an auxotroph that requires vitamins which include pyridoxine, riboflavin, pantothenic acid, and nicotinic acid. Although previous studies have reported that LB broth containing 30 g l^-1^ NaCl (modified LB) can provide the complete cell growth requirements for efficient astaxanthin production for this bacterium [27], such medium containing complex organic nitrogen sources, including tryptone and yeast extract, was not suitable for producing nitrogen-limiting and excess carbon conditions in this bacterium.

### Bioinformatic Prediction of the PHAs Biosynthesis Pathway in *P. haeundaensis*

**Fig. 2:**
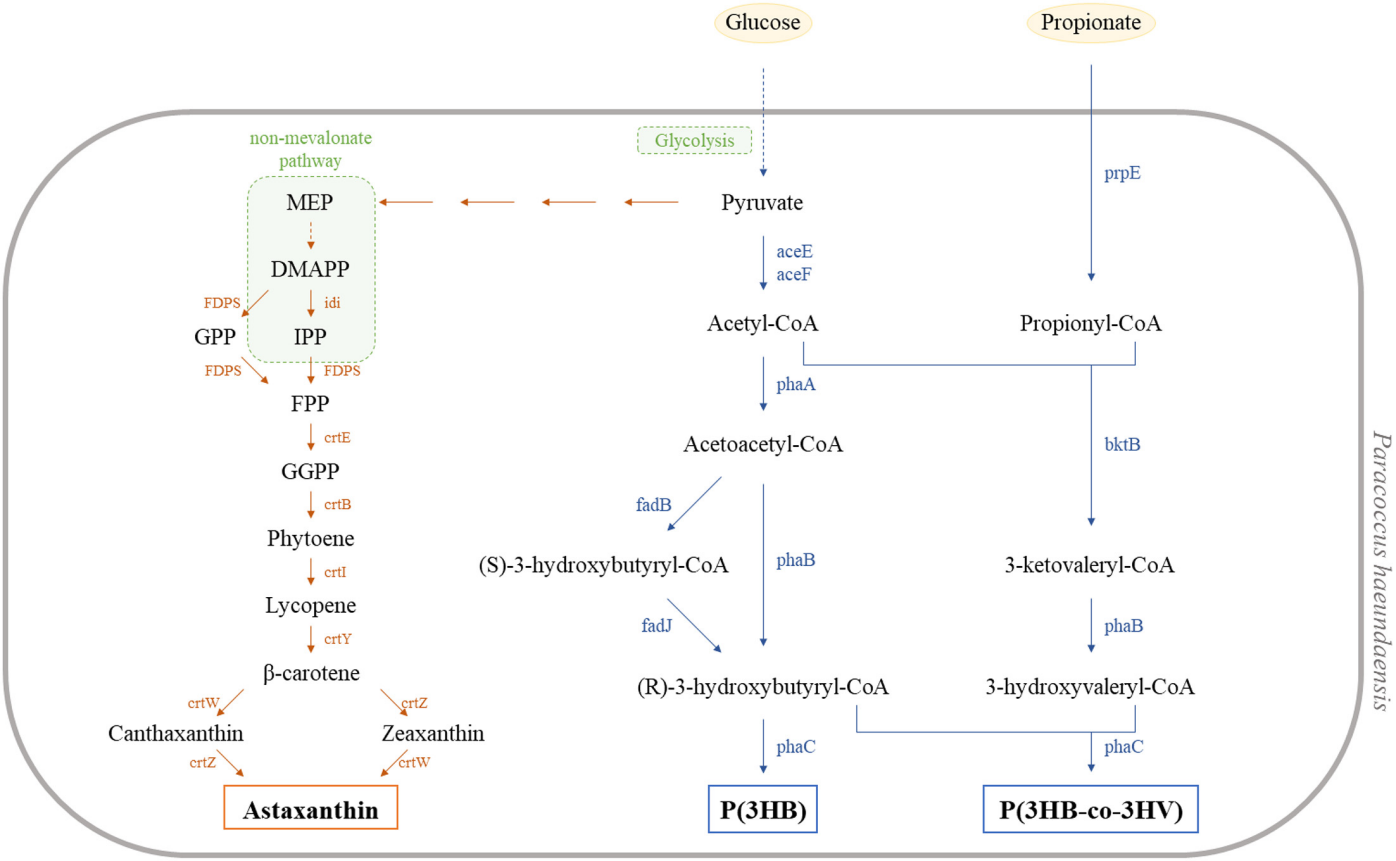
Metabolic routes proposed for PHA and astaxanthin biosynthesis in *P. haeundaensis*. MEP: 2-C-Methyl-D-erythritol 4-phosphate; DMAPP: Dimethylallyl pyrophosphate; IPP: Isopentenyl pyrophosphate; GPP: Geranyl pyrophosphate; FPP: Farnesyl pyrophosphate; GGPP: Geranylgeranyl pyrophosphate; P(3HB): Poly-3-Hydroxybutyrate; P(3HB-*co*-3HV): Poly(3-hydroxybutyrate-*co*-3-hydroxyvalerate); idi: isopentenyl diphosphate isomerase; FDPS: farnesyl diphosphate synthase; crtE: geranylgeranyl diphosphate synthase; crtB: 15-cis-phytoene synthase; crtI: phytoene desaturase; crtY: lycopene betacyclase; crtW: beta-carotene 4-ketolase; crtZ: beta-carotene 3-hydroxylase; aceE: pyruvate dehydrogenase E1 component; aceF: pyruvate dehydrogenase E2 component; phaA: acetyl-CoA C-acetyltransferase; phaB: acetoacetyl-CoA reductase; phaC: poly[(R)-3-hydroxyalkanoate] polymerase; fadB: 3-hydroxybutyryl-CoA dehydrogenase; fadJ: 3-hydroxybutyryl-CoA epimerase; prpE: propionyl-CoA synthetase; bktB: beta-ketothiolase.

The genome sequence of *P. haeundaensis*, obtained from Genbank (assembly accession: GCA_006239215.1), was used to predict the biosynthesis pathway of PHAs. Fig. 2 shows a suggested PHAs production pathway based on the corresponding enzymes present in the genome of this bacterium. Among the metabolic pathways obtained through BlastKOALA, the poly(3-hydroxybutyrate) [P(3HB)] biosynthesis pathway used in *P. haeundaensis* was predicted by comparing amino acid similarities with the enzymes and proteins involved in the P(3HB) synthesis pathway from other known P(3HB)-producing bacteria such as *Paracoccus denitrificans*, *Cupriabividus necator*, and *Chromobacterium* sp. USM2. Pyruvate is generated via glycolysis from glucose and PHAs biosynthesis starts from the C3 intermediate pyruvate. Pyruvate is then further converted into acetyl-coenzyme (acetyl-CoA) by pyruvate dehydrogenase (WP_011015247.1). Two molecules of acetyl-CoA, generated from pyruvate, are condensed by β-ketothioloase (PhaA: WP_139598808) to become acetoacetyl-CoA. This intermediate is then further reduced by nicotinamide adenine dinucleotide (NADH)-dependent acetoacetyl-CoA reductase (PhaB: TNH37875.1) and subsequently converted into (R)-3-hydroxybutyl-CoA as a P3HB precursor. The P(3HB) monomer generated through this pathway is then finally used as a substrate by PHA polymerase (PhaC: TNH40935.1) to polymerize the form of a P(3HB) polymer. In addition, to produce P(3HB-*co*-3HV) often common precursor of 3HV such as propionic acid is subsequently converted into 3-ketovaleryl CoA by β-ketothioloase. Then, condensation of one acetyl-CoA molecule with one of propionyl-CoA results in formation of 3-ketovaleryl-CoA and then proceeding through the classical route for synthesis, (R)-3-hydroxyvaleryl-CoA is synthesized. The 3-ketovaleryl CoA is finally converted into 3-hydroxvaleryl CoA by PhaB and used as a substrate by PHA polymerase (PhaC: TNH40935.1) to polymerize the form of a P(3HB-*co*-3HV). Generally, the type of PHA varies depending on the type of monomer used by the PhaC enzyme as a substrate. PhaC enzymes are classified into four classes based on their substrate specificities, primary structure as deduced from amino acid sequences, and subunit composition [28]. To predict the enzymatic function and substrate usage of PhaC in *P. haeundaensis*, its amino acid sequence homology was analyzed and compared with other known class I PhaC enzymes from PHA producing bacteria (Table S1). Among the bacteria studied for PHAs production, the PhaC enzyme from *P. haeundaensis* had the highest identity (62.92%) to PhaC from *Paracoccus denitrificans*. Several studies have reported that *Paracoccus denitrificans* produces poly(3-hydroxybutyrate) [P(3HB)] and P(3HB-*co*-3HV) [29]. This analysis indicates that *P. haeundaensis* is likely to produce both P(3HB) and P(3HB-*co*-3HV).

### PHA and Astaxanthin Production from Glucose during the Growth of *P. haeundaensis*

**Fig. 3:**
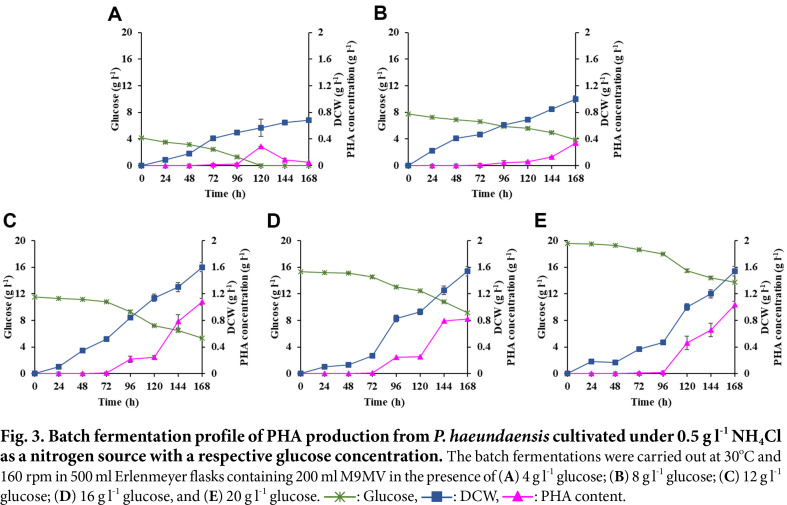


**Fig. 4:**
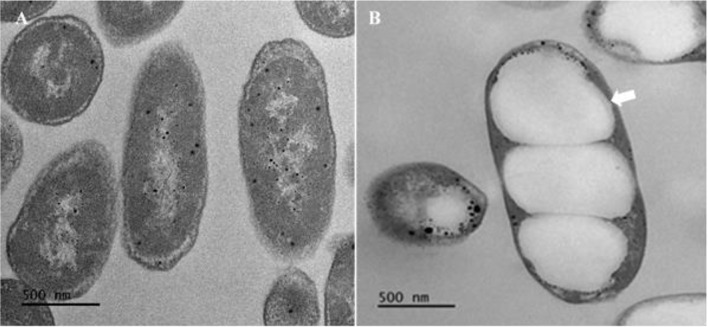
Transmission electron microscopic images (TEM) of intracellular PHA granules (white arrow) in *P. haeundaensis*. (**A**) cells from modified LB (**B**) cells from M9MV supplemented with 12 g l^-1^ of glucose and 0.5 g l^-1^ of NH_4_Cl.

To introduce *P. haeundaensis* into excess carbon and nitrogen-limited conditions for the production of PHAs, glucose was used as a basic model carbon source. For this purpose, predetermined glucose concentrations ranging from 4 g l^-1^ to 20 g l^-1^ with nitrogen fixed at a 0.5 g l^-1^ NH_4_Cl concentration were supplemented into M9MV broth. The results, which are shown in Fig. 3, show that the amount of glucose supplemented into the media dramatically affected cell biomass and PHAs concentrations. In particular, in the presence of 4 g l^-1^ glucose, this bacterium completely consumed the glucose within a 120-h cultivation time with the final cell biomass concentration increased up to 0.68 g l^-1^ during a 168-h cultivation time (Fig. 3A). During the cultivation time, the total PHAs concentration reached a maximum of 0.29 g l^-1^ within 120-h. After a 120-h cultivation time, the concentrations of PHAs decreased significantly to 0.05 g l^-1^ due to glucose depletion in the medium, indicating that PHAs were degraded by this bacterium for use as energy and carbon sources. In the presence of 8 g l^-1^ glucose, *P. haeundaensis* consumed only 4 g l^-1^ of glucose in the medium. The biomass and PHAs concentrations were increased to 1 g l^-1^ and 0.34 g l^-1^, respectively, at 168-h culture (Fig. 3B). However, in the presence of 12 g l^-1^ or higher concentrations of glucose, all final cell biomass and PHAs concentrations remained relatively constant at approximately 1.6 g l^-1^ for cell biomass and an average value of 0.89 g l^-1^ for PHA concentration during a 168-h culturing time (Fig. 3C-3E). A possible explanation for this result was the lack of an available nitrogen source for cell growth. A further increase in glucose concentration to 20 g l^-1^ did not affect the biomass concentration and the highest amount of unconsumed glucose remained in the medium, indicating that nitrogen-limited conditions were achieved for PHA synthesis by this bacterium. This agreed with the results of the analysis of the kinetic parameters for PHA production which were obtained from the batch experiments for a 168-h culturing time, as summarized in Table 1. In the presence of 12 g l^-1^ of glucose with 0.5 g l^-1^ NH_4_Cl, the highest levels of cell biomass at 1.6 g l^-1^, total PHA content at 0.63 g per g of dry cell weight (DCW) (g g^-1^), and PHA concentration at 1.03 g l^-1^ were achieved. Furthermore, we conducted experiments to explore the impact of various culture conditions, including temperature (ranging from 25°C to 35°C) and pH (from 6.0 to 10.0), on cell biomass and PHA production. Regrettably, these experiments did not yield any significant improvements in either cell biomass or PHA concentrations, as depicted in Fig. S1. To validate this result, we subjected cells harvested from the batch experiment to transmission electron microscopy (TEM) analysis, as shown in Fig. 4. The TEM image demonstrated that these conditions indeed facilitate efficient PHA accumulation in this bacterium. Consequently, we have determined that the highest PHA production from *P. haeundaensis* can be achieved when using 12 g l^-1^ of glucose as the sole carbon source under nitrogen-limited conditions, employing 0.5 g l^-1^ of NH_4_ and maintaining at 30°C and pH 8.0.

In a relevant reference concerning astaxanthin production, the pathway associated with this bacterium has been previously documented by other studies [27]. Therefore, we also explored the potential for producing this value-added product in the context of batch fermentation under nitrogen-limited conditions for PHA production. Our results revealed that 1.04 mg l^-1^ of astaxanthin can be co-produced from this bacterium under these conditions (as detailed in Fig. S2). Comparing this result to the astaxanthin titer from LB medium, this bacterium yielded a significantly lower amount of this value-added compound (5.77 mg l^-1^) without PHA production under nitrogen-rich conditions at the same temperature and pH, which were agreeable results shown in Fig. 4. This outcome indicates the competitive relationship between PHA and astaxanthin production, given that both bioproducts rely on acetyl-CoA as an initial substrate. To enhance the PHA production potential of this bacterium, it is essential to target the enzymes involved in non-mevalonate pathway responsible for astaxanthin production. Similar findings have been reported in previous studies [30].

### Identification of PHA Types Produced by *P. haeundaensis*

To determine the quality and quantity of PHA produced by *P. haeundaensis*, GC and GC/MS analyses were carried out using the cells harvested from all previous batch fermentation experiments. A typical example of a total ion chromatogram from GC/MS analysis, using a sample from the batch experiments performed in the presence of 12 g l^-1^ of glucose and 0.5 g l^-1^ ammonium concentrations, is shown in Fig. S3. The result showed several sharp peaks representing 3-hydroxybutylate methyl ester B, 3-hydroxyvalerate methyl ester C, and benzoate methyl ester as the internal standard, as shown in Fig. S3. The peaks appeared at 2.537 min and 3.587 min retention time (RT) in a total ion chromatogram (TIC), were respectively detected as 3-hydroxybutyrate methyl ester (3HBME) and 3-hydroxyvalerate methyl ester (3HVME) as electron ionization mass spectra was compared to the GC-MS library search report [Fig. S3(B) and S3(C)]. The peak that appeared at a 2.537 min was matched to a 3-hydroxybutyric acid-methyl ester with a molecular weight of 118 and a spectral match index (SI) value of 95. The peak that appeared at a 3.587 min RT was matched to a 3-hydroxyvaleric acid-methyl ester with molecular weights of 132 and an SI of 90. Based on the GC/MS analysis, this bacterium produced two different types of PHAs depending on the concentrations of glucose used as a single carbon source under nitrogen-limited conditions (Table 1). Although *P. haeundaensis* predominantly produced a homopolymer of poly(3-hydroxybutyrate) in the presence of glucose concentrations ranging from 4 g l^-1^ to 8 g l^-1^, in particular in the presence of glucose concentrations at 12 g l^-1^ or higher, this bacterium synthesized co-polymers of P(3HB-*co*-3HV) composed of 97%3-hydroxybutyrate and 3% 3-hydroxyvalerate. Therefore, we concluded that *P. haeundaensis* has a great potential to produce copolymer P(3HB-*co*-3HV) from a single carbon source concentration at 12 g l^-1^ under the nitrogen-limited conditions.

### Effect of Other Carbon Sources on the Production of Copolymer P(3HB-*co*-3HV)

Since the highest cell biomass and PHA concentrations were achieved in the presence of 12 g l^-1^ of glucose, this concentration was selected to evaluate the potential production of copolymer P(3HB-*co*-3HV) from other supplemented carbon sources. A total of seven carbon sources were chosen based on fermentable sugars from cheap raw materials such as sucrose and fructose which can be found abundantly in cane molasses from the food industry, glycerol from biodiesel byproducts, xylose derived from the main component of hemicellulose present in agricultural residue from lignocellulosic materials, galactose from cheese whey generated from the cheese manufacturing industry, and maltose from rice-residue.

**Fig. 5:**
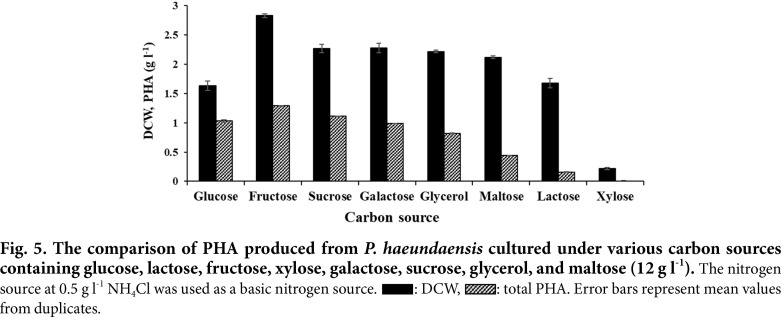


As can be seen in Fig. 5, fructose was the most preferred carbon source to produce PHA by this bacterium. The highest final cell biomass concentrations at 2.8 g l^-1^ and the highest total PHA concentration at 1.29 g l^-1^ were obtained from fructose and produced by this bacterium after a 168-h culture time. Except for xylose, all tested fermentable sugars were efficiently utilized as a carbon and energy source by *P. haeundaensis*. As determined using the molar ratios of 3HB:3HV, the PHAs mostly produced from the tested carbon sources were the copolymer types of P(3HB-*co*-3HV), except that of P(3HB) produced from maltose. In terms of the range of carbon usage and preference, the results were in agreement with other reports [31].

To evaluate the PHA production characteristics of this bacterium, we have compiled data on several well-known wild-type PHA-producing strains, such as *C. necator*, and other halophilic bacteria like *Halomonas* sp., which are detailed in Table 3. In terms of cell biomass and PHA concentrations, this bacterium yielded lower quantities compared to those from other PHA producers due to the employed fed-batch or cell retention cultivation methods. However, concerning PHA content, the nitrogen-limited conditions applied to this bacterium demonstrated significant potential, with accumulations of PHA up to 62% (wt%) as glucose was used as a single carbon source. Furthermore, widely recognized PHA-producing strains, including *C. necator* and *Halomonas*, predominantly synthesized homopolymer-type PHA (PHB) when single carbon sources were used. In contrast, *P. haeundaensis* strain produced copolymer types P(3HB-*co*-3HV) [PHBV] from unrelated single carbon sources. Among the compared strains, only *Paracoccus* sp. LL1 showed comparable characteristics.

From a commercial perspective, PHBV exhibits more favorable properties than PHB [32]. Additionally, most organisms require a 3HV precursor, such as propionic acid, for PHBV synthesis. However, *P. haeundaensis* can synthesize PHBV from unrelated 3HV precursors. This is particularly advantageous from a commercialization standpoint, as the substrate cost accounts for 40-60% of the total PHA production cost [33], and pure propionic acid, when used as a co-substrate for PHBV, tends to be relatively expensive. Thus, utilizing a single carbon source for PHBV production can significantly reduce production costs. However, further development of media and fermentation methods using a fed-batch approach would further enhance critical parameters, including cell biomass titer and PHA productivities.

### Effect of Propionic Acid as a Secondary Carbon Source on PHAs Production

Previous studies have frequently employed short-chain fatty acids to diversify the range of PHAs produced by various bacteria, resulting in the synthesis of different copolymer types of P(3HB-*co*-3HV) with higher molar ratios of 3HV [28, 29, 34]. Our *in-silico* prediction analysis also suggested that this bacterium can utilize propionic acid as a secondary substrate for the production of P(3HB-*co*-3HV) copolymers (Fig. 2).

To investigate the effect of propionic acid as a secondary carbon source, we conducted experiments with concentrations ranging from 0.5 g l^-1^ to 4 g l^-1^ in the presence of 12 g l^-1^ of glucose in M9MV media. As shown in Table 4, *P. haeundaensis* produced various copolymers of P(3HB-*co*-3HV) with different molar ratios of 3HV in response to the supplementation of varying amounts of propionic acid. Specifically, in the presence of 3 g l^-1^ of propionic acid and 12 g l^-1^ of glucose, this bacterium produced a copolymer P(3HB-*co*-3HV) with a molar ratio of 50% 3HB and 50% 3HV. However, when more than 1 g l^-1^ of propionic acid was added, the precursor not only inhibited cell growth, but also affected the total PHA content. The results can be explained as follows: When a short-chain fatty acid like propionic acid is supplied as a precursor for the 3-hydroxyvalerate monomer unit of PHA, the available acyl-CoA molecules, including acetyl-CoA and propionyl-CoA generated by propionyl-CoA synthetase (prpE, as shown in Fig. 2) are increased. Particularly, under nitrogen-limited conditions, the enzymes involved in tricarboxylic acid cycle (TCA) processing of both acyl-CoA molecules are inhibited for *de novo* synthesis, which leads to a reduction in cell biomass production. Consequently, the remaining acyl-CoA molecules, not fully utilized in the TCA cycle, enter the PHA synthesis pathway. In the PHA synthesis pathway, additional acetyl-CoA and propionyl-CoA are utilized to synthesize 3-ketovaleryl CoA by beta-ketothiolase (bktB, as shown in Fig. 2), which is further converted into 3-hydroxyvaleryl-CoA. Ultimately, these additional monomer units of 3-hydroxyvaleryl-CoA contribute to the synthesis of copolymer types of P(3HB-*co*-3HV) with higher molar ratios, facilitated by poly[(R)-3-hydroxyalkanoate] polymerase (phaC, as shown in Fig. 2). Similar results have also been reported in other studies [14, 35].

## Conclusion

This study highlights the PHA production capability of *Paracoccus haeundaensis* and was performed under small-scale batch mode conditions by the control of the carbon and nitrogen sources concentrations which induced both PHA and astaxanthin production in this bacterium. In particular, it was demonstrated that *P. haeundaensis* is an auxotroph requiring pyridoxine, riboflavin, pantothenic acid, and nicotinic acid. From this view, an efficient fully defined media, M9MV, was developed for this bacterium to provide not only efficient cell growth, but also introduce nitrogen-limited conditions for efficient PHAs production. Of note, when 12 g l^-1^ of glucose with a small amount of inorganic nitrogen at 0.5 g l^-1^ of NH_4_Cl was added into the M9MV medium, *P. haeundaensis* produced the copolymer type PHA, P(3HB-*co*-3HV) with the maximum concentration of cell biomass at 1.6 g l^-1^ and PHA at 1.03 g l^-1^ with the highest total PHA content at 0.64 g per g of dry cell weight and the coproduction of 1.04 mg l -1 astaxanthin. The types of PHA produced from *P. haeundaensis* were investigated using PHA synthesis pathway prediction based on genome information and batch fermentation experiments. It was demonstrated that this bacterium can produce the copolymer P(3HB-*co*-3HV) composed of 97% 3-hydroxybutyrate (3HB) and 3% 3-hydroxyvalerate (3HV) under nitrogen-limited conditions using a single carbon source concentration at 12 g l^-1^ or higher. In addition, this bacterium can produce various copolymers of P(3HB-*co*-3HV) with different molar ratios of 3HV in response to the supplementation of varying amounts of propionic acid used as a secondary carbon source. The results also showed that fructose was the most efficient and preferred carbon source and produced up to 2.8 g l^-1^ of cell biomass and 1.29 g l^-1^ of PHA with 0.46 g of PHA per g of dry cell weight. *P. haeundaensis*, a marine halophilic bacterium, not only has potential advantages in reducing cost by a cheaper carbon source used for PHA production, but also produces astaxanthin, which can be used as a value-added product. However, further studies are required related to fed-batch mode experiments to increase PHA yields and productivities. In addition, the wild type strain *P. haeundaensis* needs to be further improved to increase propionic acid resistance, using random mutagenesis or genetic improvements.

## Supplemental Materials

Supplementary data for this paper are available on-line only at http://jmb.or.kr.



## Figures and Tables

**Table 1:** Batch fermentation kinetics of *P. haeundaensis* for PHA production after a 168-h culture time.

Glucose (g l^-1^)	Biomass (g l^-1^)	3HB content (g g^-1^)	3HV content (g g^-1^)	3HB:3HV molar ratio (%)	PHA (g l^-1^)
4	0.7 ± 0.03	0.08 ± 0.006^a^	N/D^b^	100:0	0.05
8	1.0 ± 0.05	0.36 ± 0.027	N/D	100:0	0.34
12	1.6 ± 0.08	0.61 ± 0.090	0.02 ± 0.014	97:3	1.03
16	1.5 ± 0.08	0.47 ± 0.044	0.02 ± 0.020	96:4	0.75
20	1.6 ± 0.08	0.56 ± 0.079	0.02 ± 0.022	97:3	0.90

The kinetic parameters were analyzed from batch fermentation carried out in 500 ml Erlenmeyer flasks containing a 200 ml working volume using respective glucose concentrations in the presence of 0.5 g l^-1^ NH_4_Cl as a nitrogen source at 30°C and 160 rpm.

^a^Mean values generated from triplicates

^b^Not Detected

**Table 2:** The comparison of PHA production kinetics from *P. haeundaensis*.

Carbon source	Biomass (g l^-1^)	3HB content (g g^-1^)	3HV content (g g^-1^)	3HB:3HV molar ratio (%)	PHA (g l^-1^)
Glucose	1.6 ± 0.08^a^	0.61 ± 0.090	0.01 ± 0.014	97:3	1.03
Fructose	2.8 ± 0.03	0.44 ± 0.004	0.01 ± 0.001	98:2	1.29
Sucrose	2.2 ± 0.07	0.48 ± 0.002	0.01 ± 0.020	98:2	1.11
Galactose	2.2 ± 0.08	0.43 ± 0.010	0.01 ± 0.001	98:2	0.99
Glycerol	2.2 ± 0.02	0.35 ± 0.005	0.01 ± 0.001	98:2	0.82
Maltose	2.1 ± 0.02	0.20 ± 0.008	N/D	100:0	0.44
Lactose	1.6 ± 0.08	0.08 ± 0.004	0.01 ± 0.021	94:6	0.16
Xylose	0.2 ± 0.02	N/D^b^	N/D	N/D	N/D

The kinetic parameters were analyzed from batch fermentation carried out in 500 ml Erlenmeyer flasks containing a 200 ml working volume using respective carbon sources at 12 g l^-1^ in the presence of 0.5 g l^-1^ NH_4_Cl as a nitrogen source at 30°C, pH 8.0 and 160 rpm.

^a^Mean values generated from duplicates

^b^Not Detected

**Table 3:** The comparison of PHA production from *Paracoccus haeundaensis* with other PHA producing strains.

Strain	Substrate	Mode of cultivation	PHA type	Biomass (g l^-1^)	PHA (g l^-1^)	PHA contents (wt %)	Astaxanthin (mg l^-1^)	References
Terrestrial producer								
*Cupriavidus necator* DSM 545	Glucose	Batch	P (3HB)	4.84	2.38	49.1	N/A^a^	[36]
*Methylobacterium organophilum*	Methanol	Fed-batch	P (3HB)	250	130	52	N/A	[37]
*Ralstonia eutropha*	Glucose	Fed-batch	P (3HB)	221	180	82	N/A	[38]
*Pseudomonas extorquens*	Methanol	Fed-batch	P (3HB)	233	149	64	N/A	[39]
Halophilic bacteria								
*Halomonas boliviensis*	Glucose	Fed-batch	P (3HB)	44.0	35.4	81	N/A	[40]
*Halomonas bluephagenesis* TD01	Glucose	Batch	P (3HB)	8.61	N/D^b^	70.45	N/A	[32]
*Halomonas campisalis* MCM B-1027	Maltose	Batch	P (3HB)	1.72	0.97	56.23	N/A	[41]
*P. denitrificans* PD01	n-Pentanol	Batch	P (3HB)	1.1	0.55	49	N/A	[42]
*Paracoccus* sp. LL1	Glycerol	Fed-batch	P(3HB-*co*-HV)	24.2	9.52	39.3	1.0	[30]
	Glycerol	Batch	P(3HB-*co*-HV)	1.3	0.4	34.5	0.8	[30]
	Glucose	Batch	P(3HB-*co*-HV)	19.2	6.97	36.3	1.9	[34]
	Glucose	Fed-batch	P(3HB-*co*-HV)	113	43.9	38.8	8.51	[34]
*Paracoccus haeundaesis*	Glucose	Batch	P(3HB-*co*-HV)	1.6	1.03	62	1.04	This study
	Fructose	Batch	P(3HB-*co*-HV)	2.8	1.29	46.07	N/D	This study

^a^Not Applicable

^b^Not Determined

**Table 4:** The comparison of PHAs production using propionic acid as a secondary carbon source.

Propionic acid (g l^-1^)	Biomass (g l^-1^)	3HB content (g g^-1^)	3HV content (g g^-1^)	Total PHA content (g g^-1^)	3HB:3HV mol (%)	PHA (g l^-1^)
0	1.6 ± 0.08^a^	0.63 ± 0.090	0.02 ± 0.014	0.65	97:3	1.03
0.5	2.0 ± 0.10	0.39 ± 0.005	0.01 ± 0.002	0.40	98:2	0.79
1	1.7 ± 0.08	0.21 ± 0.001	0.01 ± 0.001	0.22	95:5	0.38
2	1.4 ± 0.07	0.05 ± 0.002	0.03 ± 0.002	0.08	67:33	0.10
3	1.2 ± 0.06	0.02 ± 0.001	0.02 ± 0.001	0.04	50:50	0.05
4	0.9 ± 0.04	0.02 ± 0.001	0.02 ± 0.001	0.04	53:47	0.04

^a^Mean values generated from duplicates
