# Supplementary material for: The Halophilic Bacterium Paracoccus haeundaensis for the Production of Poly(3-Hydroxybutyrate-co-3-Hydroxyvalerate) from Single Carbon Sources
Source: J Microbiol Biotechnol. 2023 Nov 13;34(1):74–84. doi: 10.4014/jmb.2305.05025 (PMC10840474; doi:10.4014/jmb.2305.05025)
Supplement: Supplementary file 1 [file jmb-34-1-74-supple.pdf]

https://doi.org/10.4014/jmb.2305.05025

Supplementary Table and Figures

Table 1S. Amino acids homology comparison of class I PHA synthase and the type of PHA from *P. haeundaensis.*

Bacteria	PhaC		Product PHA	Ref.
	Identity (%)	Accession no.		
P. marcusii	99.09	WP_217844568	[a]N/A	N/A
P. denitrificans	62.92	BAA77257.1	[b]P(3HB), P(3HB-co-3HV)	[25]
C. necator	32.04	QQB78658.1	P(3HB), P(3HB-co-4HB)	[28]
Chromobacterium sp. USM2	31.39	ADL70203.1	P(3HB), P(3HB-co-3HH), P(3HB-co-3HV-co-3HH)	[29]
Streptomyces aureofaciens	16.74	AAK53451.1	P(3HB)	[30]

[a]N/A: Not available.

[b]P(3HB): Poly(3-hydroxybutyric acid); P(3HB-co-3HV): Poly(3-hydroxybutyric acid-co-3-hydroxyvaleric acid); P(3HB-co-4HB): Poly(3-hydroxybutyric acid-co-4-hydroxybutyric acid); P(3HB-co-3HH): Poly(3-hydroxybutyric acid-co-3-hydroxyhexanoic acid).

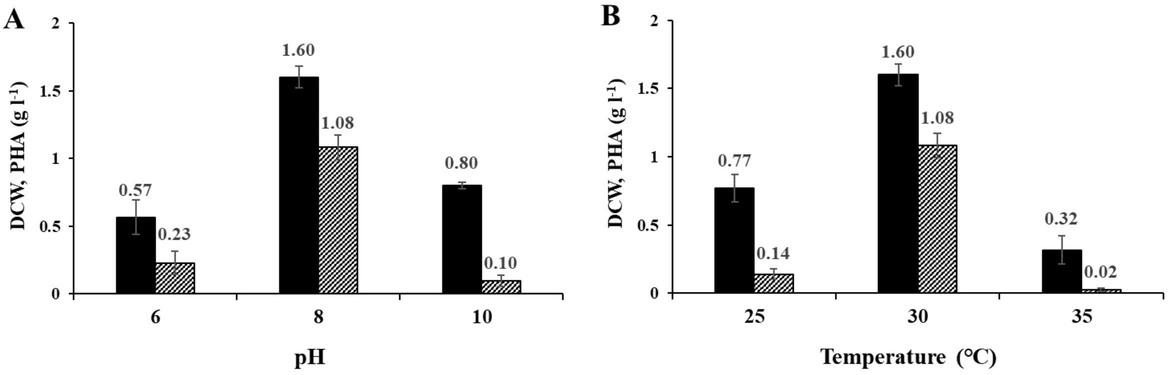

Fig. 1S. The comparison of PHA produced from *P. haeundaensis* cultured under various pH and temperatures. **(A)** The comparison of biomass and PHA production from *P. haeundaensis* at pH 6, 8, and 10. **(B)** The comparison of biomass and PHA production from *P. haeundaensis* at temperature 25, 30 and 35(°C) : DCW, : total PHA. Error bars represent mean values from duplicates.

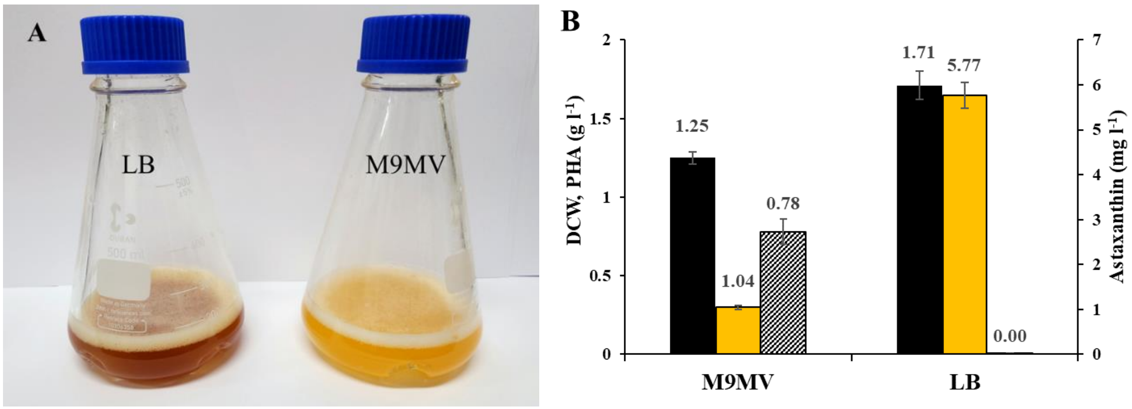

Fig. 2S. The comparison of PHA and astaxanthin from *P. haeundaensis* under nitrogen-rich (LB) and nitrogen-limited conditions (M9MV). (**A**) The comparison of *P. haeundaensis* cultures color change observed by astaxanthin production in LB medium and M9MV medium. (**B**) The comparison of PHA and astaxanthin production in *P. haeundaensis* cultured in LB medium and M9MV medium. : DCW, : total PHA, : Astaxanthin. Error bars represent mean values from duplicates.

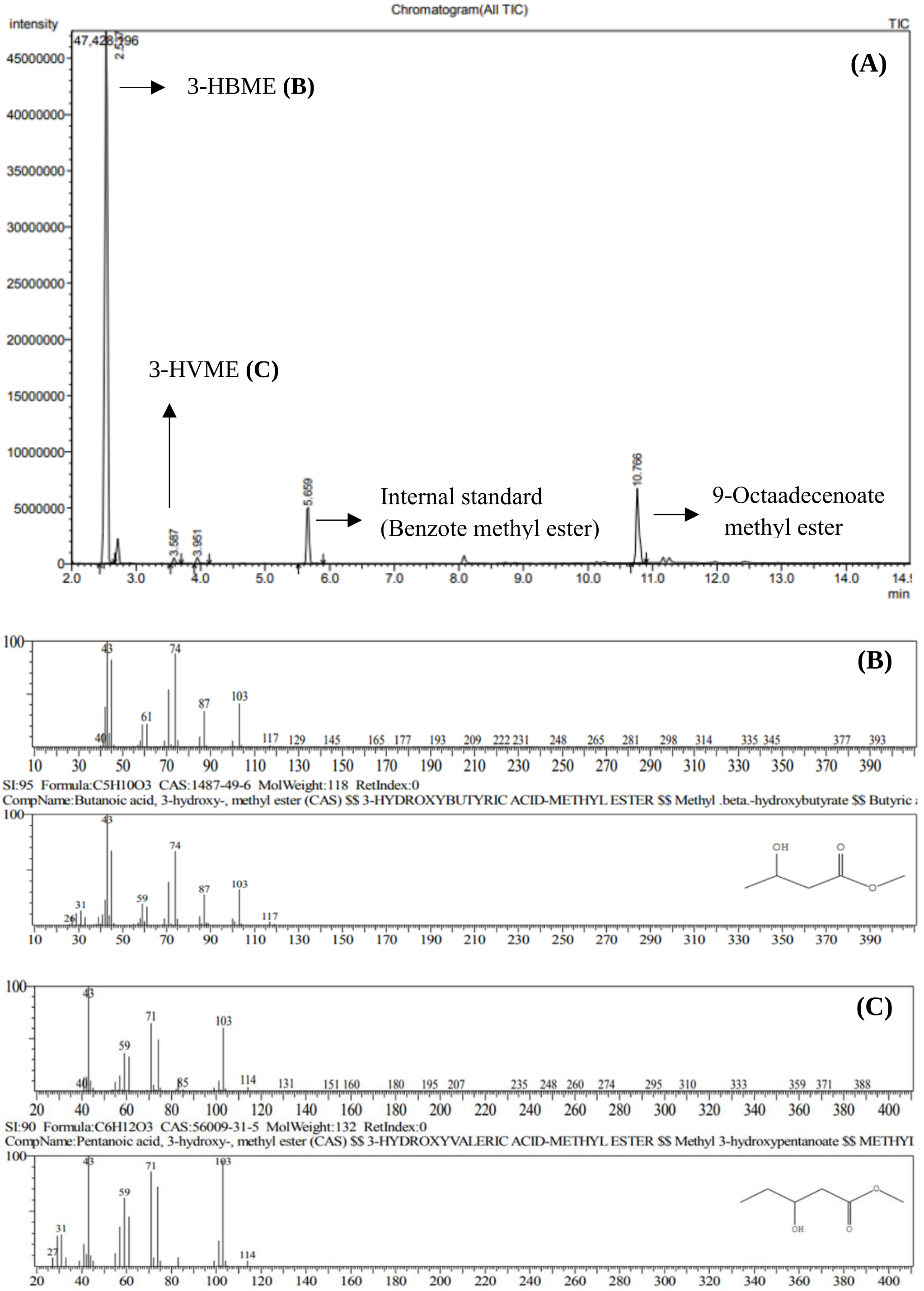

Fig. 3S. GC-MS results of PHA extracted from *P. haeundaensis* cells cultivated in M9MV supplemented with 12 g·L^{-1} glucose and 0.5 g·L^{-1} NH_4Cl and harvested at 168 h. The sample was analyzed using methanolysis method. **(A)** Total ion chromatogram (TIC) of PHA sample, **(B)** Electron ionization mass spectrum of 3-hydroxybutyl methyl ester(3HBME) detected from sample, **(C)** Electron ionization mass spectrum of 3-hydroxyvalerate methyl ester(3HVME) detected from sample.
